# Biological Evaluation and Molecular Dynamics Simulation of Chalcone Derivatives as Epidermal Growth Factor-Tyrosine Kinase Inhibitors

**DOI:** 10.3390/molecules24061092

**Published:** 2019-03-20

**Authors:** Kanyani Sangpheak, Lueacha Tabtimmai, Supaphorn Seetaha, Chompoonut Rungnim, Warinthorn Chavasiri, Peter Wolschann, Kiattawee Choowongkomon, Thanyada Rungrotmongkol

**Affiliations:** 1 Program in Biotechnology, Faculty of Science, Chulalongkorn University, Bangkok 10330, Thailand; swaratchada@gmail.com; 2 Department of Biochemistry, Faculty of Science, Kasetsart University, Bangkok 10903, Thailand; Ahcaeul@gmail.com (L.T.); supaporn.se@ku.th (S.S.); kiattawee.c@ku.th (K.C.); 3 National Nanotechnology Center (NANOTEC), National Science and Technology Development Agency (NSTDA), Pathum Thani 12120, Thailand; chompoonut@nanotec.or.th; 4 Center of Excellence in Natural Products Chemistry, Department of Chemistry, Faculty of Science, Chulalongkorn University, Bangkok 10330, Thailand; warinthorn.c@chula.ac.th; 5 Department of Pharmaceutical Chemistry, University of Vienna, Vienna 1090, Austria; karl.peter.wolschann@univie.ac.at; 6 Institute of Theoretical Chemistry, University of Vienna, Vienna 1090, Austria; 7 Structural and Computational Biology Research Unit, Department of Biochemistry, Faculty of Science, Chulalongkorn University, Bangkok 10330, Thailand; 8 Ph.D. Program in Bioinformatics and Computational Biology, Faculty of Science, Chulalongkorn University, Bangkok 10330, Thailand

**Keywords:** chalcone derivatives, cytotoxicity assay, EGFR tyrosine kinase, molecular dynamics simulation, ADMET

## Abstract

Targeted cancer therapy has become a high potential cancer treatment. Epidermal growth factor receptor (EGFR), which plays an important role in cell signaling, enhanced cell survival and proliferation, has been suggested as molecular target for the development of novel cancer therapeutics. In this study, a series of chalcone derivatives was screened by in vitro cytotoxicity against the wild type (A431 and A549) and mutant EGFR (H1975 and H1650) cancer cell lines, and, subsequently, tested for EGFR-tyrosine kinase (TK) inhibition. From the experimental screening, all chalcones seemed to be more active against the A431 than the A549 cell line, with chalcones **1c**, **2a**, **3e**, **4e**, and **4t** showing a more than 50% inhibitory activity against the EGFR-TK activity and a high cytotoxicity with IC_50_ values of < 10 µM against A431 cells. Moreover, these five chalcones showed more potent on H1975 (T790M/L858R mutation) than H1650 (exon 19 deletion E746-A750) cell lines. Only three chalcones (**1c**, **2a** and **3e**) had an inhibitory activity against EGFR-TK with a relative inhibition percentage that was close to the approved drug, erlotinib. Molecular dynamics studies on their complexes with EGFR-TK domain in aqueous solution affirmed that they were well-occupied within the ATP binding site and strongly interacted with seven hydrophobic residues, including the important hinge region residue M793. From the above information, as well as ADMET (absorption, distribution, metabolism, excretion, and toxicity) properties, all three chalcones could serve as lead compounds for the development of EGFR-TK inhibitors.

## 1. Introduction

Cancer is the second leading cause of death globally, with an estimated 9.6 million deaths due to cancer in 2018, or about 1 in 6 deaths worldwide [1]. Currently, chemotherapy is a widely used and often highly effective cancer treatment. However, chemotherapeutic agents have severe side effects and sometimes they have a limited selectivity for tumor cells over normal cells, resulting in systemic toxicity and the appearance of drug-resistant tumor cells [2]. Hence, it is necessary to develop new and more efficient drugs, with no or diminished side effects and a greater selectivity against cancer. Targeted therapy is considered as a more efficient treatment due to its specificity towards cancer cells. Targeted cancer therapies use drugs to block the growth of cancer by interfering with molecular targets. They can attack a specific target in tumors by disrupting the cell growth process with less damage to normal cells.

**Figure 1:**
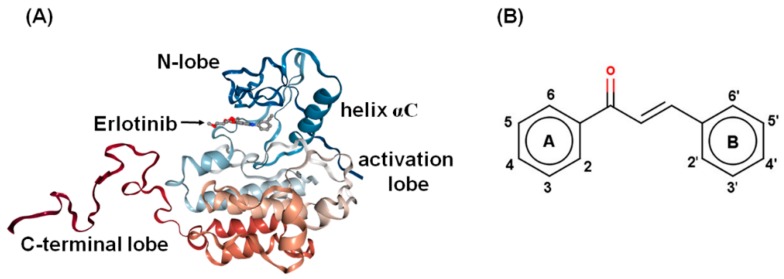
(**A**) Three-dimensional structure of the epidermal growth factor receptor tyrosine kinase (EGFR-TK) domain in the active conformation with erlotinib bound, as modeled from the crystal structure (PDB: 1M17), and (**B**) the chemical structure of natural chalcone, where its synthesized derivatives are taken from a previous study [39].

The epidermal growth factor (EGFR; Erb1 or HER1) is one of four transmembrane proteins in the family of transforming growth factor receptors. It has been suggested as a candidate for targeted cancer therapy [3,4], since it plays an important role in cell signaling, enhanced cell survival, proliferation, and resistance to anti-cancer therapeutics. It is highly expressed in human epidermoid carcinoma A431 cells and human non-small lung cancer cells [5,6,7,8]. Its tyrosine kinase (TK) domain is shown in Figure 1A. The clinically available drugs used as a tyrosine kinase inhibitor of EGFR (EGFR-TKI), include inhibitor gefitinib [9], erlotinib [10], and lapatinib [11], plus the irreversible inhibitors afatinib [12] and dacomitinib [13]. 

Chalcones, or 1,3-diphenyl-2-propene-1-ones (Figure 1B), are in the class of agents that have shown a promising therapeutic efficacy against several types of cancer and also display characteristics of an EGFR inhibitor [14,15,16]. They consist of two aromatic rings connected by a linker of a three-carbon α, β-unsaturated carbonyl system. The chalcone core structure is one of the most important intermediate and essential features of a variety of flavonoids and medicinal agents. Natural and synthetic chalcone derivatives demonstrate a broad spectrum of therapeutic effects, such as anti-inflammatory, anti-microbial, anti-bacterial, anti-fungal, anti-viral, anti-oxidant, and, especially, anti-cancer activities [17,18,19,20,21,22,23,24]. The anti-cancer potentials of chalcones and their derivatives have been reported in a wide range of cancer cell lines, comprised of breast (MCF-7) [25], lung (A549) [26,27], cervical (HeLa) [26], liver (HEP-2) [28], and colon (HT-29) cancers [29,30]. 

Chalcones have attracted attention because of their promising therapeutic effects, as they are able to target multiple cellular molecules, such as MDM2/p53, tubulin, proteasome, NF-kappa B, TRIAL/death receptors and mitochondria mediated apoptotic pathways, cell cycle, Signal transducer and activator of transcription 3, Activator protein 1, nuclear erythroid 2-related factor 2, Peroxisome proliferator-activated receptor gamma [31], human topoisomerase IIα [32,33,34,35], and EGFR-TK. The hybrid of triazoloquinoxaline-chalcone derivatives showed dual EGFR-TKI and tubulin polymerization inhibition activities [36]. A series of novel tetrahydro-[1,2,4]triazolo[3,4-a] isoquinolin-3-yl)-3-arylprop-2-en-1-one derivatives revealed that the trimethoxy groups of potent chalcones may be responsible for the higher levels of EGFR and dihydrofolate reductase inhibition [37]. Moreover, a series of novel chalcone derivatives were found to increase the level of reactive oxygen species in MCF-7 cells, eventually leading to apoptosis through intrinsic, as well as extrinsic pathways [38].

In the present study, in order to find new potential anti-cancer agents against EGFR-TK, a series of 47 previous synthesized chalcone derivatives [39] were screened in vitro for cytotoxicity towards two cancer cell lines with wild type EGFR expression derived from a human epidermoid carcinoma (A431) and human lung adenocarcinoma (A549), and two human lung cancer cell lines with EGFR mutants (H1975 and H1650) using the surrogate 3-(4,5-dimethylthiazole-2-yl)-2,5-diphenyltetrazolium bromide (MTT) assay. The chalcones which showed a high cytotoxic effect against the cancer cell lines were then tested for their TKI effects on recombinant (*r*)EGFR. Then, all-atom molecular dynamics simulations were performed to investigate the structure and dynamic properties, as well as the ligand-target interactions between the most potent chalcones and the EGFR-TK target.

## 2. Results and Discussion

### 2.1. Cytotoxicity Effect Against the Wild Type (A431 and A549) and Mutant EGFR (H1650 and H1975) Cancer Cell Lines

**Figure 2:**
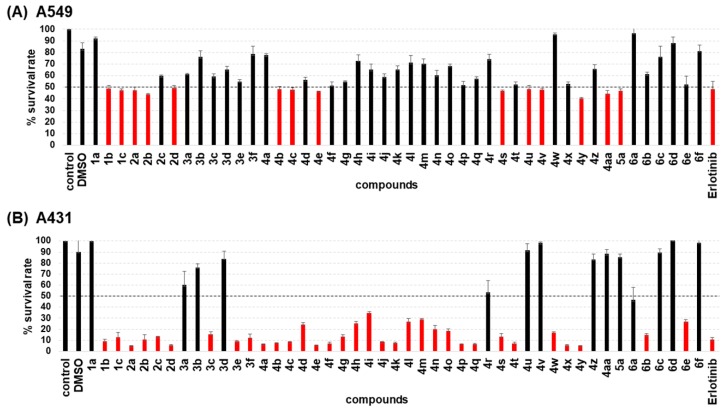
Relative survival rate (%) of the (**A**) A549 and (**B**) A431 cells after treatment with 100 µM of the indicated chalcone derivative or erlotinib for 72 h. Data are shown as the mean ± one standard deviation, derived from three independent repeats of triplicate cultures. Means in red bars are ≤ 50% survival (below the black dashed line) and those with a different letter are significantly different (*p* < 0.05).

The 47 synthesized chalcone compounds (**1a**–**6e**) from a previous study [39] and the approved anti-cancer drug (erlotinib and afatinib) as an EGFR-TKI were evaluated for their in vitro cytotoxic activity against two wild type EGFR, human epidermoid carcinoma (A431) and lung adenocarcinoma (A549), and two mutant EGFR human lung cancer cell lines (H1650 with exon 19 deletion E746-A750 and H1975 with T790M/L858R mutation) using the MTT assay. Preliminary results (not shown) established a concentration of 100 μM was suitable to test the chalcone derivatives and erlotinib, and the number of viable cells (survival rate) relative to the untreated cells (%) of the two wild type EGFR cancer cells are plotted in Figure 2. Some of the chalcone derivatives exhibited promising cytotoxic effects against both cancer cell lines, with a resulting relative cell viability of less than 50% (red bar). This series of 47 chalcone derivatives seemed to inhibit A431 cells better than A549, which may reflect the different expression levels of the EGFR [40,41], since A431 cells have a high level of EGFR expression and, so, are more sensitive to EGFR-inhibiting agents than A549, which has a lower EGFR expression level. Moreover, the KRAS (K-ras) mutation found in A549 cell lines is able to alter the downstream signaling pathway of EGFR. Importantly, the KRAS mutation is linked to the primary resistance of EGFR-TKIs [42]. Therefore, inhibition of the EGFR may not be able to completely inhibit the proliferation of the A549 cancer cell line.

After preliminary screening, the 36 compounds that demonstrated a ≥50% reduction in cell viability at a concentration of 100 µM were then selected for evaluating the half maximal inhibitory concentration (IC_50_) values. The derived IC_50_ values of the focused chalcones and erlotinib on the two cancer cell lines are summarized in Table 1. All 36 chalcones showed moderate to good anticancer activity with IC_50_ values in the range of 5.0−55.0 μM against A431, whereas they displayed moderate to poor activity on the A549 cell line. The five compounds which exhibited the highest level of cytotoxicity were **4t**, **1c**, **2a**, **4e**, and **3e** with IC_50_ values of 5.0 ± 3.5, 8.0 ± 1.2, 9.9 ± 4.9, 10.0 ± 5.8 and 10.5 ± 7.4 µM against the A431 cell line, respectively. The IC_50_ of erlotinib on A431 and A549 was 0.6 ± 0.1 and 18.8 ± 2.4 µM. Considering the data from the in vitro screening of cytotoxicity against cancer cell lines, it is possible that the chalcone derivatives tend to inhibit the high level of EGFR expression in A431 cells. This is in good agreement with previous studies in which the cytotoxicity of Ec-LDP-hBD1 to A431 cells (high EGFR expression cells) was more potent than that to the lung carcinoma A549 and H460 cell lines with a low EGFR expression level [8]. These focused chalcones were then tested on the two additional cell lines, H1650 and H1975, and their derived IC_50_ values are presented in Table 1. Afatinib was used for the positive control. It can be seen that they were less effective in the H1650 cells (IC_50_ of 9.2–23.8 μM) as compared to the H1975 cell line (IC_50_ of 5.1–17.8 μM), somewhat similar to shikonin, the main active component of Zi Cao [43,44,45]. However, it seems that our potent chalcones were more effective with the wild type EGFR A431 cell lines than the two mutant EGFR cancer cell lines. 

It is worth noting that the series of chalcones used in this study showed no toxicity to human embryonic fibroblast (HEF) cells (Figure S1, Supporting Information). However, to gain additional information about the inhibition of EGFR at the TK domain by the five potent chalcones, their in vitro EGFR-TKI activity was evaluated against the intracellular domain (ICD) of the EGFR and compared with erlotinib. 

### 2.2. EGFR-TKI Activity by Chalcones 

**Figure 3:**
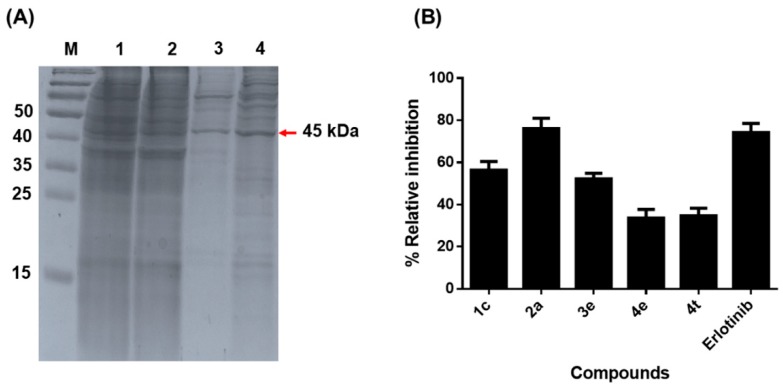
(**A**) Representative sodium dodecyl sulfate-polyacrylamide gel electrophoresis (SDS-PAGE) gel analysis of the enrichment of the *r*EGFR-TK intracellular domain (ICD), loading 5 µg protein per track. Lane M: molecular weight marker of standard protein; Lane 1: supernatant, Lane 2: Flow through, Lane 3: 10% of buffer B, Lane 4: 45 kDa of EGFR-TK domain (**B**) The relative *r*EGFR-TKI activity (%) of the five potent chalcone derivatives and erlotinib at 1 μM, as assayed using the ADP-glo kinase assay. Data are shown as the mean ± one standard deviation, derived from three independent repeats. Means with a different letter are significantly different (*p* < 0.05).

In order to assess the EGFR-TKI activity of erlotinib and the five potent chalcone derivatives (**1c**, **2a**, **3e**, **4e**, and **4t**), the intracellular domain of *r*EGFR-TK was expressed in a mammalian expression system so as to retain the full kinase activity [46]. The *r*EGFR-TK domain appeared to be a major band with approximate size around 45 kDa kinase domain following sodium dodecyl sulfate-polyacrylamide gel electrophoresis (SDS-PAGE) resolution and Coomassie blue staining (Figure 3A). The EGFR-TKI activity of the five potent chalcones and erlotinib at 1 μM was then comparatively studied using a commercial kit (ADP-Glo™ Kinase Assay), and the obtained relative inhibition level (%) is shown in Figure 3B. The chalcones **1c**, **2a**, and **3e** showed a relative EGFR-TKI activity of more than 50%, while the other two compounds (**4e** and **4t**) seemed to be less effective against this enzyme. Interestingly, chalcone **2a** with the highest EGFR-TKI activity (76.2%) was slightly higher than that for erlotinib (75.1%). Then, the three chalcones that showed the EGFR-TK inhibition more than 50% (**1c**, **2a**, and **3e**) were then selected for evaluating the half maximal inhibitory concentration (IC_50_) values relative to erlotinib (see also, Table 1). The three chalcones exhibited an EGFR-TKI activity with IC_50_ in range 10.3—15.4 μM, while erlotinib inhibited EGFR-TK with an IC_50_ 24.29 nM. Although, these three derivatives did not provide the inhibitory activity better than erlotinib, they still showed as the promising agents inhibiting the EGFR-TK. To investigate the detailed binding and interaction of these three potent chalcones (**1c**, **2a**, and **3e**) against the EGFR-TK domain, the ligand-protein complexes were then studied using MD simulations in aqueous solution.

### 2.3. Molecular Binding and Interaction of Potent Chalcones 

**Figure 4:**
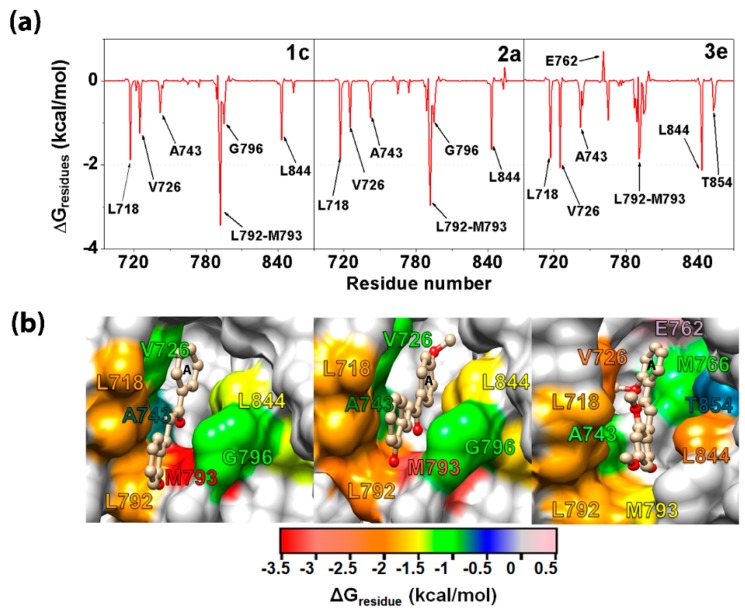
(**A**) Per-residue decomposition free energy of the three chalcone/EGFR-TK complexes and (**B**) their binding orientation inside the ATP-binding pocket of the TK domain drawn from the last MD snapshot, where the energy contour of residue contribution for ligand binding is shaded.

**Figure 5:**
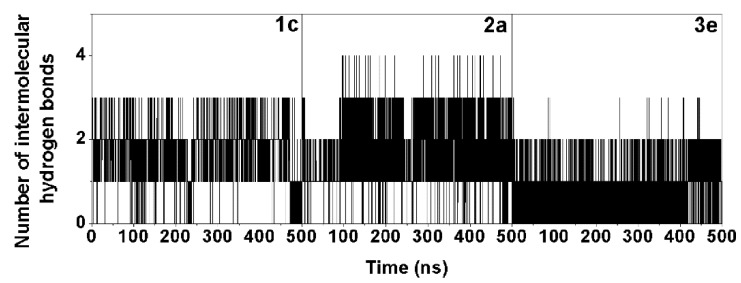
Time evolution of the number of intermolecular hydrogen bonds (H-bonds) formed between the EGFR-TK residues and the three screened chalcones.

The 500-ns MD simulations were performed in triplicate on each complex of the three selected chalcones (**1c**, **2a**, and **3e**) binding with the EGFR-TK domain at the ATP binding site. The energy fluctuation curves and RMSD of each simulation were shown in Supplemental Figures S2 and S3. Since the chalcone binding pattern and intermolecular interactions with EGFR-TK obtained from the three independent simulations were relatively similar, the results presented here are taken from one representative simulation. To find the key residues of EGFR-TK for chalcone binding, the per-residue decomposition free energy (ΔG_residue_) based on the MM/GBSA method was applied on the 100 snapshots over the last 100-ns simulation. Among residues 695–1,018 of the EGFR-TK (Figure 1A), only the results for residues 695–870 are plotted in Figure 4A, where the ligand binding orientation inside the ATP-binding pocket of EGFR-TK with the contour energy of residue contribution is illustrated in Figure 4B. Note that the negative and positive ΔG_residue_ values indicated the stabilization and destabilization energies for ligand binding, respectively. In addition, the number of the hydrogen bonds (H-bonds) of the three chalcones was computed over the simulation period (Figure 5).

From Figure 4B, all three potent chalcones (**1c**, **2a**, and **3e**) shared a similar orientation in the EGFR-TK ATP binding site, in which the aryl moiety (A ring in Figure 1B) was deeply inserted into and the carbonyl oxygen pointed towards the binding pocket, which is in accordance with a previous computational study on other chalcone analogs [16]. These three compounds (**1c**, **2a**, and **3e**) were preferentially stabilized by the seven EGFR residues: L718, V726, A743, L792, M793, G796, and L844 with an energy contribution of −0.5 kcal/mol (Figure 4A). The residue T854 (−0.78 kcal/mol) additionally contributed to the **3e** binding in compensation with a destabilization by E762 (0.70 kcal/mol). This implies that these residues play a significant role in the binding of **1c**, **2a**, and **3e** to the TK domain of the EGFR. Some chalcone binding residues observed in this work, such as L718, A743, L792, M793, G796, and L844, were also found in a major interaction between erlotinib and the EGFR in the co-crystallized 1M17 structure [47,48]. Among the three considered chalcones, **2a** formed the strongest H-bond interactions with the target enzyme (Figure 5). According to the MD simulation results, three or four H-bonds are formed with the M793, T790, and T854 residues. The two H-bonds formed with the hinge region residue M793 were also found in **1c** and **3e**, which could explain the importance of M793 as it provided a relatively high stabilization for these three focused chalcones. For example, changing from this –OH group at the R_2_ position on B ring (**2a**) to the –OCH_3_ group (**2c**) has significantly reduced the anti-cancer activity (Table 1). In addition, this H-bond formation with M793 was previously reported as the main interaction in erlotinib analogues and gefitinib in complex with wild type and mutant EGFR strains [49,50,51]. Moreover, it was suggested that M793 plays a significant role in interacting with various EGFR inhibitors including erlotinib [52].

**Figure 6:**
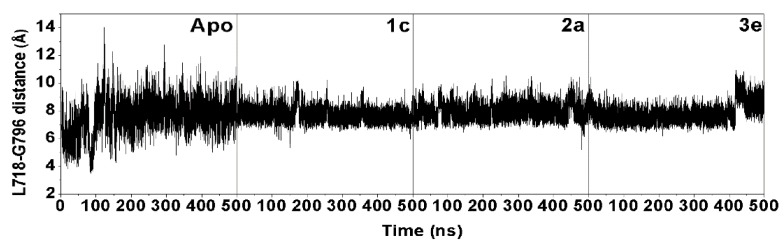
Time-dependent distance between the centers of mass of residues L718 and G796 for the apo and complex forms of the EGFR over a 500 ns MD trajectory.

**Figure 7:**
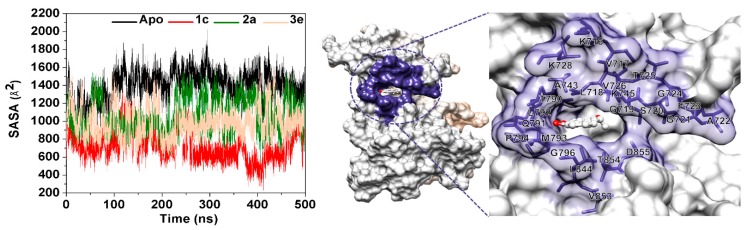
Solvent-accessible surface area (SASA, Å^2^) of the apo form and three chalcone complexes along the 500-ns MD where the amino acids within a 7-Å sphere of chalcone used for the SASA calculations are also shown on the right-hand side.

To identify the structural change in the ATP binding pocket affected by complexation with chalcones, the distance between the centers of mass of the two hydrophobic residues (L718 and G796; Figure 4B) was calculated during the MD simulation time. The distance plot (Figure 6) showed that in comparison to the apo system (~4.0 to ~14.0 Å) the three complexes had a shorter distance (~7.0 to ~9.0 Å), which was maintained at a lower fluctuation level, indicating an induced-fitted mechanism for chalcone binding to EGFR-TK. This result was well supported by the lower water accessibility into the ATP binding site (Figure 7) in all three complexes (**1c**: ~400–1200 Å^2^; **2a**: ~800–1500 Å^2^; and **3e** ~700–1500 Å^2^) relative to that for the apo form (~1300–2000 Å^2^). Note that increase in L718-G796 distance in the system **3e** after 400 ns had affected to ligand binding pocket only as seen by increased RMSD of the binding residues by 0.5 Å without overall structure change (Supplemental Figure S3).

Based on all the above data, these three chalcone derivatives could potentially serve as new candidates for anti-cancer drug development against the EGFR. 

### 2.4. Physicochemical Properties of the Potent Chalcones

The physicochemical and pharmacokinetic properties, as well as the toxicity of the three focused chalcones, were investigated by the absorption, distribution, metabolism, excretion, and toxicity (ADMET) prediction using the online SwissADME web program (www.swissadme.ch/) [53], Molinspiration cheminformatics (http://www.molinspiration.com/) web program [54], and Osiris prediction tool on DataWarrior program [55]. Their toxicity risk (mutagenicity, tumorigenicity, irritation, and reproduction), physicochemical (molecular weight, partition coefficient (cLog*P*), total polar surface area, and solubility), drug likeness, and pharmacokinetic (gastrointestinal absorption, blood–brain barrier permeant, P-glycoprotein substrate, and cytochrome P450 inhibitor) properties are summarized in Table 2. From the toxicity prediction, the active derivatives **1c**, **2a** and **3e** had no predicated adverse risk of mutagenicity, tumorgenicity, irritating effects and reproductive effects. For the physicochemical properties, they showed a moderate solubility, while erlotinib was poorly soluble. Thus, erlotinib requires high doses in order to reach therapeutic plasma concentrations after oral administration. The pharmacokinetic profiles of all compounds suggested that they could be CYP2C19 and CYP2C9 inhibitors. By considering the drug likeness, the three potent chalcones could likely be used as an orally active drug for human.

## 3. Materials and Methods 

### 3.1. Materials and Measurements

Epidermoid carcinoma (A431; CRL-1555) and human lung adenocarcinoma (A549; CCL-185) express EGFR derived cell lines were obtained from the American Type Cell Culture Collection, USA, while the two EGFR mutated human lung cancer cell lines (H1975 and H1650) were provided by Dr. Chanida Vinayanuwattikun from Department of Medicine, Chulalongkorn University and HEF cells from the Ramathibodi hospital. Dulbecco’s modified Eagle’s medium (DMEM), fetal bovine serum (FBS), penicillin-streptomycin (Pen-Strep), and trypsin were purchased from Life Technologies (Carlsbad, CA, USA). Thiazolyl blue (MTT), dimethyl sulfoxide (DMSO), SDS and phosphate buffer saline were purchased from Sigma-Aldrich (Darmstat, Germany). The ADP-GloTM Kinase Assay kit was purchased from Promega (Madison, WI, USA). The series of chalcone derivatives were synthesized at the Center of Excellence in Natural Products Chemistry, Chulalongkorn University [39]. All solvents and reagents used for synthesis were purchased from Sigma-Aldrich (St. Louis, MO, USA), Merck (Kenilworth, NJ, USA), or TCI chemical companies (Tokyo, Japan). 

### 3.2. Cell Culture and Cell Viability Assay (MTT Assay)

The in vitro cytotoxicity activities of the chalcone derivatives against the A431, A549, H1975 and H1650 cell lines were evaluated using the MTT reduction assay as a surrogate marker of the relative number of viable cells. The A431, A549, HEF cells were grown in complete DMEM medium (CM; DMEM supplemented with 10% (v/v) FBS, 100 U/mL penicillin and 100 U/mL streptomycin) while H1975 and H1650 were grown in complete RPMI-1640 medium at 37 °C in a 5% (v/v) CO_2_, 95% (v/v) air humidified incubator. For preliminary screening, 100 μL of A431 (5000 cells/well), A549 (5000 cells/well), and HEF (7000 cells/well) cell suspension was seeded per well in a 96-well microplate and incubated at 37 °C overnight. The medium was then changed for fresh CM containing the respective test compounds or erlotinib (positive control) at 100 µM and incubated for 72 h. The media was then added with 10 μL of MTT solution and incubated for 3 h before being removed and replaced by 50 µL of DMSO to lyse the cells and solubilize the formazan crystals prior to measurement of the absorption at 570 nm and 630 nm wavelengths using a microplate reader (Infinite M200 micro-plate reader, Tecan, Männedorf, Switzerland). 

After screening, the compounds that reduced the relative cell viability to less than 50% of the control were selected for determining their IC_50_ value. The potent chalcones against the wild type of EGFR cancer cell lines were then tested with the EGFR mutated human lung cancer cell lines H1975 and H1650. The relative survival rate and IC_50_ were analyzed using GraphPad Prism version 6.0. Each experiment was performed in triplicate and repeated three times. Note that the cytotoxicity of these 47 chalcone derivatives against HEF cells was also tested by the same method. 

### 3.3. Enrichment of the ICD of rEGFR from Transfected Hela Cells

The EGFR expression plasmid, pcDNA6A-EGFR ICD (645-1186) was a gift from Mien-Chie Hung (Addgene plasmid # 42667) [46]. The plasmid was transfected into the expression host HeLa cells using the DarmaFECT reagent according to manufacturer’s instruction. Twenty-four h after transfection, the transfected HeLa cells were selected using BlasticidinS-HCl (Invitrogen) for one month. The cells were then cultured in five T75 cm^2^ flasks for protein extraction. When the cells reached 90% confluency they were harvested and extracted using RIPA buffer. The ICD-EGFR was then enriched by anion-exchange column (GigaCap Q) chromatography (Tosoh Bioscience, Tokyo, Japan) using AKTA Primer Plus FPLC with buffer A (50 mM Tris-HCl, pH 8.0, 50 mM NaCl, 1 mM mercaptoethanol, 5 mM MgCl_2_, 1 mM EDTA, and 5% (v/v) glycerol) for equilibration and buffer B (as per Buffer A except 1 M not 50 mM NaCl) for elution. The *r*EGFR (ICD) protein enrichment was monitored by 12% (w/v) acrylamide resolving gel SDS-PAGE. Protein concentration was calculated by measuring the absorbance at 280 nm, using ε (280) = 52,370 M^−1^ cm^−1^ for EGFR.

### 3.4. EGFR-TKI Assay

The selected chalcones (**1c**, **2a**, **3e**, **4e**, and **4t**), those with a cytotoxic IC_50_ of less than 10 μM, plus erlotinib were screened for their ability to inhibit the tyrosine kinase activity of the enriched rEGFR using the ADP-Glo™ Kinase Assay. The first 8 µL of buffer (40 mM Tris-HCI pH 7.5, 20 mM MgCl_2_, 0.1 mg/mL bovine serum albumen) was added to the wells of a 384-well plate (Promega, solid white). Then, 5 µL of 2 ng/µL of enzymes and 1 µM of the respective inhibitor was added to triplicate wells, followed by 10 µL of a mixture of 5 µM ATP and 2.5 μM poly(glu·tyr), and incubated for 1 h at room temperature. Next, 5 μL of the ADP-Glo reagent was added, incubated for 40 min and then 10 µL kinase detection reagent was added and incubated at room temperature for 30 min to convert the ADP to ATP. The ATP was then detected by measuring the luminescence using a microplate spectrophotometer system (Synergy HTX Multi-Mode reader, BioTek, Winooski, VT, USA). After screening, the compounds that inhibited EGFR-TK more than 50% were selected for determining their IC_50_ value. All assays were performed in triplicate. The relative inhibition (%) of erlotinib and the chalcone derivatives was then calculated compared to that of the no-inhibitor control. 

### 3.5. Molecular Dynamics Simulation

We employed homology modeling to generate the full structure of the TK domain from the crystal structure of the EGFR-TK domain (pdb code: 1M17) [51] as the template and using the SWISS-MODEL program. From the in vitro cytotoxicity studies, the selected chalcones were then docked into the ATP binding site of the EGFR-TK model using the CDOCKER module [56], in accordance with the standard procedures [56,57]. The docked chalcone/EGFR-TK complex with lowest interaction energy was selected as the initial structure for performing the MDSs. The partial charges of each ligand were prepared as follows. The geometry of ligand was optimized with ab initio calculation using the HF/6-31G* method in the Gaussian 09 Revision E.01 program [58]. Its electrostatic potential (ESP) charges were evaluated using the same level of theory, and then the restrained ESP charges were retrieved by the charge fitting procedure using the antechamber module in AMBER 16 [59]. The ligand and protein were treated by the general AMBER force field (GAFF) [60] and AMBER ff14SB force field [61], respectively. The protonation states of the Asp, Glu, Lys, Arg, and His residues were assigned using PROPKA 3.0 [62]. The complex was solvated by TIP3P water molecules within 12 Å around the system surface, and chloride ions were randomly added for system neutralization.

All missing hydrogen atoms were added and then minimized with 1000 steps of steepest descents (SD) and 2000 steps of conjugated gradients (CG) using the Sander module in AMBER 16 to reduce the bad contacts and steric hindrance. The water molecules and ions were then minimized with 2500 steps of SD followed by 2500 steps of CG, while the protein backbone was restrained with a force constant of 10.0, 5.0 and 1.0 kcal/mol·Å, respectively. Finally, the whole system was fully minimized with 2500 steps of SD and CG. All covalent bonds involving hydrogen atoms were constrained by the SHAKE algorithm [63]. The long-range electrostatic interactions were calculated by the Particle Mesh Ewald (PME) approach [64], while a cutoff distance of 12 Å was applied for non-bonded interactions. Each system was heated to 310 K for 200 ps and equilibrated at the same temperature for 200 ps with backbone restraints by a force 10 kcal/mol·Å. After that, 1 ns simulation was performed by decreasing the restraint weights on protein from 10.0 to 5.0 kcal/mol·Å and then the simulation without any restraint was conducted till 500 ns. The MD trajectories from the last 100-ns simulation were taken for analysis in terms of root mean square deviation, per-residue decomposition free energy, number of H-bonds between the ligand and EGFR-TK, time-dependent distance between the centers of mass of the hydrophobic residues L718 and G796, and the water accessibility as the SASA. 

## 4. Conclusions

A series of 47 synthesized chalcone derivatives were in vitro tested for their cytotoxicity to two wild type EGFR (A431 and A549) and two mutant EGFR (H1650 and H1975) cancer cell lines by the MTT assay in comparison with erlotinib and afatinib, as known inhibitors for wild type and mutant EGFR-TK, respectively. All chalcones seemed to be more effective against A431 than A549, which may reflect the lower expression levels of the EGFR and the mutation of KRAS (K-ras) on A549 cell lines. Thus, blocking the EGFR TK activity by our chalcones may not be able to completely inhibit the A549 cancer cell proliferation. The five most active chalcones (**1c**, **2a**, **3e**, **4e**, and **4t**) showed the highest in vitro cytotoxicity against the A431 cancer cell lines. They were more effective on H1975 cell lines than H1650. However, it seems that these compounds presented higher inhibitory activity against the wild type EGFR (A431) than the two mutant EGFR cancer cell lines. All chalcones showed no toxicity to HEF cells, while they were more effective with the high-level EGFR expression cancer cell lines. It is possible that our chalcones are likely to be effective only against cancers involving EGFR activation, since there are 2x10^6^ EGFR receptors on the cancer cell surface with 1000-fold higher than normal cells. Only **1c**, **2a**, and **3e** inhibited the EGFR TK activity more than 50%. These three chalcone derivatives in complex with EGFR-TK were selected to study the inhibition mechanism at a molecular level by all-atom MD simulation in aqueous solution. From the MD simulation results, the key residues responsible for chalcone binding were L718, V276, A743, L972, M973, G976, and L844, while **3e** was additionally stabilized by T854 in compensation with E762 destabilization. The hinge region residue M793 exhibited the strongest energy stabilization through H-bonding, as previously found for other known EGFR-TKIs. In summary, the in silico and in vitro results suggested that three chalcone derivatives (**1c**, **2a**, and **3e**) can potentially serve as lead compounds for further anti-cancer drug development. 

## Figures and Tables

**Table 1:** Derived in vitro cytotoxicity IC_50_ values of the potent chalcone derivatives against the A431, A549, H1650, and H1975 cell lines and wild type EGFR-TK compared to erlotinib and afatinib.

Compound	IC_50_ value (μM) Against:				IC_50_ Against EGFR-TK
	A431	A549	H1650	H1975	
**1b**	34.0 ± 7.38	50.9 ± 3.8	-	-	-
**1c**	8.0 ± 1.2	25.4 ± 1.2	13.1 ± 2.8	9.2 ± 2.3	10.3 μM
**2a**	9.9 ± 4.9	20.2 ± 1.9	10.0 ± 0.7	5.1 ± 0.3	13.8 μM
**2b**	29.5 ± 3.5	69.4 ± 7.6	-	-	-
**2c**	24.6 ± 6.0	>100	-	-	-
**2d**	26.6 ± 6.9	25.4 ± 1.7	-	-	-
**3c**	20.7 ± 9.8	>100	-	-	-
**3e**	10.5 ± 7.4	>100	23.8 ± 2.1	14.6 ± 1.1	15.4 μM
**3f**	18.9 ± 11.1	>100	-	-	-
**4a**	38.9 ± 5.2	>100	-	-	-
**4b**	25.0 ± 8.7	44.1 ± 9.4	-	-	-
**4c**	26.6 ± 5.5	20.2 ± 1.9	-	-	-
**4d**	25.1 ± 4.3	>100	-	-	-
**4e**	10.0 ± 5.8	44.2 ± 5.3	22.2 ± 7.4	17.8 ± 1.8	-
**4f**	38.8 ± 1.6	>100	-	-	-
**4g**	21.8 ± 5.3	>100	-	-	-
**4h**	48.8 ± 3.6	>100	-	-	-
**4j**	24.0 ± 2.6	>100	-	-	-
**4k**	14.9 ± 7.6	>100	-	-	-
**4l**	29.1 ± 4.1	>100	-	-	-
**4m**	55.0 ± 6.7	>100	-	-	-
**4n**	22.0 ± 5.3	>100	-	-	-
**4o**	21.7 ± 6.8	>100	-	-	-
**4p**	37.5 ± 4.0	>100	-	-	-
**4q**	25.9 ± 3.8	>100	-	-	-
**4s**	39.5 ± 7.4	25.4 ± 2.2	-	-	-
**4t**	5.0 ± 3.5	>100	9.2 ± 0.8	6.7 ± 2.8	-
**4u**	>100	>100	-	-	-
**4v**	>100	>100	-	-	-
**4w**	24.2 ± 4.9	>100	-	-	-
**4x**	41.5 ± 6.6	>100	-	-	-
**4y**	41.5 ± 2.0	49.4 ± 7.9	-	-	-
**4aa**	>100	74.4 ± 6.5	-	-	-
**5a**	>100	>100	-	-	-
**6b**	33.4 ± 3.1	>100	-	-	-
**6e**	40.0 ± 3.9	>100	-	-	-
**Erlotinib**	0.6 ± 0.1	18.8 ± 2.4	-	-	24.29 nM
**Afatinib**	-	-	2.4 ± 0.4	1.9 ± 0.3	-

Data are shown as the mean ± one standard deviation, derived from three independent repeats or triplicate cultures. Means with a different letter are significantly different (*p* < 0.05).

**Table 2:** Absorption, distribution, metabolism, excretion, and toxicity (ADMET), drug-likeness, and pharmacokinetics of three potent chalcones.

ADMET Parameter		1c	2a	3e	Erlotinib
**Toxicity risk**	Mutation ^a^	+++	+++	+++	+++
	Tumor ^a^	+++	+++	+++	+++
	Irritant ^a^	+++	+++	+++	+++
	Reproduction effective ^a^	+++	+++	+++	+++
**Physicochemical properties**	Molecular weight (g/mol) ^b^	224.25	254.28	314.33	393.44
	cLOG*P* ^b^	2.96	2.88	2.75	2.79
	TPSA (Å^2^) ^b^	37.30	46.53	65.00	74.73
	Solubility class ^c^	Moderately soluble	Moderately soluble	Moderately soluble	Poorly soluble
**Drug likeness**	Lipinski’s rule of five ^c^	Yes; 0 violation	Yes; 0 violation	Yes; 0 violation	Yes; 0 violation
	Ghose ^c^	Yes	Yes	Yes	Yes
	Veber ^c^	Yes	Yes	Yes	Yes
	Egan ^c^	Yes	Yes	Yes	Yes
	Muegge ^c^	Yes	Yes	Yes	Yes
**Pharmacokinetic**	Gastro Intestinal absorption (%) ^c^	High	High	High	High
	Blood-brain barrier permeant ^c^	Yes	Yes	Yes	Yes
	P-gp substrate ^c^	No	No	No	No
	CYP1A2 inhibitor ^c^	No	Yes	Yes	Yes
	CYP2C19 inhibitor ^c^	Yes	Yes	Yes	Yes
	CYP2C9 inhibitor ^c^	Yes	Yes	Yes	Yes
	CYP2D6 inhibitor ^c^	No	No	No	Yes
	CYP3A4 inhibitor ^c^	No	Yes	Yes	Yes

^a^ Predicted properties taken from Osiris on Dataworrier program; +++, not toxic; ++ low toxic; - high toxic; ^b^ predicted properties taken from Molinspiration cheminformatics; ^c^ drug likeness and pharmacokinetic obtained from SwissADME.

## Supplementary Materials

The following are available online at https://www.mdpi.com/1420-3049/24/6/1092/s1, Figure S1: The percent survival rates of the 47 chalcones against human embryonic fibroblast compared with approved anticancer drugs, Figure S2: The comparison of total, potential, and kinetic energy plotted as the fluctuation curves for apo form (black), **1c** (red), **2a** (blue), and **3e** (green), Figure S3: RMSDs for all atoms for chalcone/EGFR TK complex, binding pocket residues within 7 Å of ligand, and chalcone along the 500-ns MD simulation.
