# Supplementary material for: Biological Evaluation and Molecular Dynamics Simulation of Chalcone Derivatives as Epidermal Growth Factor-Tyrosine Kinase Inhibitors
Source: Molecules. 2019 Mar 20;24(6):1092. doi: 10.3390/molecules24061092 (PMC6471738; doi:10.3390/molecules24061092)
Supplement: Supplementary file 1 [file molecules-24-01092-s001.pdf]

Supplementary Material

Biological Evaluation and Molecular Dynamics Simulation of Chalcone Derivatives as Epidermal Growth Factor-Tyrosine Kinase Inhibitors

Kanyani Sangpheak[1], Lueacha Tabtimmai[2], Supaporn Seetaha[2], Chompoonut Rungnim[3], Warinthron Chavasirid[4], Peter Wolschann[5,6*], Kiattawee Choowongkomon[2], Thanyada Rungrotmongkol[7*]

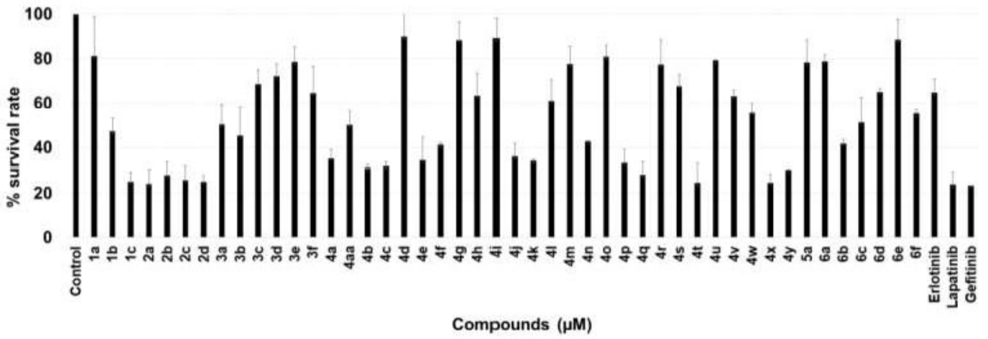

Figure S1. The percent survival rates of the 47 chalcones against human embryonic fibroblast compared with approved anticancer drugs.

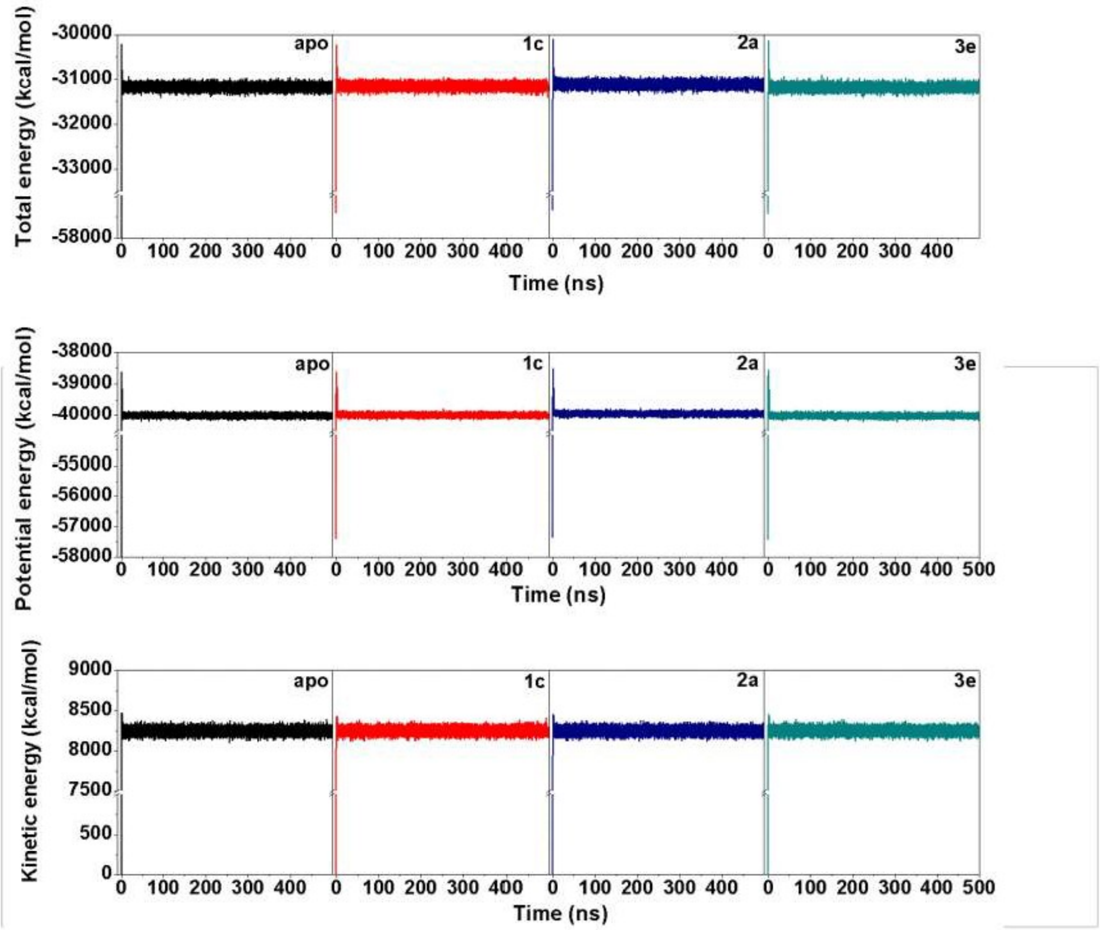

Fig. S2 The comparison of total, potential, and kinetic energy plotted as the fluctuation curves for apo form (black), **1c** (red), **2a** (blue), and **3e** (green).

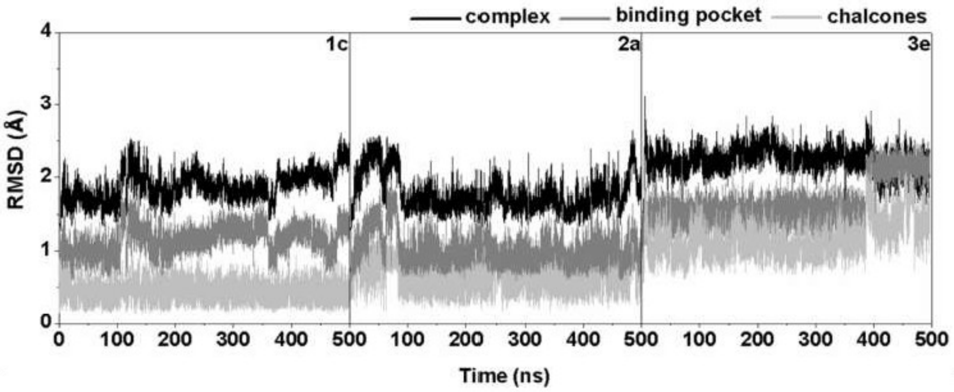

Fig. S3 RMSDs for all atoms for chalcone/EGFR TK complex, binding pocket residues within 7 Å of ligand, and chalcone along the 500-ns MD simulation
